# Bidirectional connectivity alterations in schizophrenia: a multivariate, machine-learning approach

**DOI:** 10.3389/fpsyt.2023.1232015

**Published:** 2023-09-07

**Authors:** Minhoe Kim, Ji Won Seo, Seokho Yun, Minchul Kim

**Affiliations:** ^1^Computer Convergence Software Department, Korea University, Sejong, Republic of Korea; ^2^Department of Radiology, Research Institute and Hospital of National Cancer Center, Goyang-si, Republic of Korea; ^3^Department of Psychiatry, Yeungnam University School of Medicine and College of Medicine, Daegu, Republic of Korea; ^4^Department of Radiology, Kangbuk Samsung Hospital, Sungkyunkwan University School of Medicine, Seoul, Republic of Korea

**Keywords:** resting state functional connectivity, schizophrenia, connectome-based predictive modeling, machine learning, multivariate analysis

## Abstract

**Objective:**

It is well known that altered functional connectivity is a robust neuroimaging marker of schizophrenia. However, there is inconsistency in the direction of alterations, i.e., increased or decreased connectivity. In this study, we aimed to determine the direction of the connectivity alteration associated with schizophrenia using a multivariate, data-driven approach.

**Methods:**

Resting-state functional magnetic resonance imaging data were acquired from 109 individuals with schizophrenia and 120 controls across two openly available datasets. A whole-brain resting-state functional connectivity (rsFC) matrix was computed for each individual. A modified connectome-based predictive model (CPM) with a support vector machine (SVM) was used to classify patients and controls. We conducted a series of multivariate classification analyses using three different feature sets, increased, decreased, and both increased and decreased rsFC.

**Results:**

For both datasets, combining information from both increased and decreased rsFC substantially improved prediction accuracy (Dataset 1: accuracy = 70.2%, permutation *p* = 0.001; Dataset 2: accuracy = 64.4%, permutation *p* = 0.003). When tested across datasets, the prediction model using decreased rsFC performed best. The identified predictive features of decreased rsFC were distributed mostly in the motor network for both datasets.

**Conclusion:**

These findings suggest that bidirectional alterations in rsFC are distributed in schizophrenia patients, with the pattern of decreased rsFC being more similar across different populations.

## Introduction

Schizophrenia is a severe mental illness characterized by abnormal thinking, delusions, and hallucinations. One aspect of the disease that receiving attention from researchers is the abnormal connectivity between brain regions, and ‘dysconnectivity’ is thought to be one of the pathophysiology of schizophrenia (1). Neuroimaging studies have provided evidence supporting this idea, showing abnormal functional connectivity in several networks in schizophrenia patients (2).

However, there is an inconsistency regarding whether only decreased (disconnectivity) connections, or both increased and decreased connections (dysconnectivity), exist in schizophrenia (3). Even meta-analyses have shown inconsistent results; while some previous studies have only reported decreased connectivity (2, 3), others have reported both decreased and increased connectivity (4, 5). This discrepancy can be explained by an effect of decreased network hubness in schizophrenia (6); people with schizophrenia have a reduced degree and centrality of network hub nodes, which results in higher diverse connectivity but generally decreased connectivity across the brain (6). If increased connectivity is a result of higher diversity or randomness, then it follows logically that the inconsistent discovery of increased connectivity between studies.

Existing research investigating abnormal functional connectivity in schizophrenia mostly relies on mass-univariate analysis. In contrast to mass-univariate, multivariate techniques integrate all brain features into a ‘prediction’ about the outcome, providing focused tests that avoid multiple comparisons as well as increased statistical power. (7–9). This is suitable to disclose weak, distributed effects in the brain, e.g., increased connectivity in schizophrenia (10). Further, the multivariate approach is an information-based philosophy, which focuses on the information contained in a brain and how this information may be communicated to other parts of the brain (11).

However, there is no previous study investigating the direction of connectivity alteration in schizophrenia in a multivariate manner. Connectome-based predictive modeling (CPM) is a recently developed multivariate method that has been applied to reveal data-driven associations between functional connections in the brain and clinical outcomes. Interestingly, CPM identifies both increased and decreased connectivity features separately during feature selection which is suitable for investigating the direction of connectivity alteration (12). If adding a predictor results in an accuracy increase in multivariate analysis, it means that the predictor has added information to the outcome variable (13). Thus, we implement a CPM to investigate the discrepancy in previous research. “Does increased connectivity add information regarding schizophrenia?”

## Materials and methods

### Dataset description and image acquisition

We used two openly available resting state fMRI (rsfMRI) datasets in this study.

Dataset 1 (50 patients and 120 controls), containing data acquired as part of the UCLA Consortium for Neuropsychiatric Phenomics LA5c Study 5, was obtained from the OpenfMRI database (accession number: ds000030, https://openneuro.org/datasets/ds000030/versions/1.0.0) (14). All patients underwent a semistructured assessment using the Structured Clinical Interview for DSM-IV disorders (SCID; Diagnostic and Statistical Manual of Mental Disorders, DSM-IV). Exclusion criteria included left-handedness, pregnancy, history of head injury with loss of consciousness or cognitive sequelae, and other contraindications to scanning. After receiving a verbal explanation of the study, participants gave written informed consent following procedures approved by the Institutional Review Boards at UCLA and the Los Angeles County Department of Mental Health. rsfMRI in Dataset 1 (UCLA) was acquired using two 3 T Siemens scanners. The sequence parameters were as follows: repetition time/echo time (TR/TE) = 2000/30 ms; flip angle = 90°; 34 axial slices per volume; voxel size = 3 × 3 × 4 mm^3^; and the number of volumes = 152. After receiving a verbal explanation of the study, participants gave written informed consent following procedures approved by the Institutional Review Boards at UCLA and the Los Angeles County Department of Mental Health.

Dataset 2 (70 patients and 76 controls) was provided by the Centers of Biomedical Research Excellence (COBRE).^1^ For patients in this dataset, a diagnosis of schizophrenia was made using the SCID. Exclusion criteria included confirmed or suspected pregnancy, any history of neurological disorders, and a history of intellectual disability. Written informed consent was obtained from participants according to institutional guidelines at the University of New Mexico. Dataset 2 (COBRE) was acquired using a 3 T Siemens scanner. The sequence parameters were as follows: repetition time/echo time (TR/TE) = 2000/29 ms; 32 axial slices per volume; voxel size = 3 × 3 × 4 mm^3^; and the number of volumes = 150. Written informed consent was obtained from participants according to institutional guidelines at the University of New Mexico.

To compare demographic variables, contingency *χ*^2^ tests and independent sample *t*-tests were used to examine group differences in demographics for both datasets. The statistical analyses were conducted in MATLAB 2020b.

### Image processing and patient selection

A unified functional image preprocessing pipeline was used for each dataset. The resting-state fMRI data were analyzed using the GRETNA software package (15) in SPM12 (Wellcome Department of Imaging Neuroscience, London, United Kingdom; www.fil.ion.ucl.ac.uk/spm). We used the preprocessing parameters used in our previous publication (16, 17). The first 10 volumes were discarded to allow the signal to reach equilibrium. The remaining volumes were preprocessed by slice-timing, realigning, and normalizing to the EPI template with a resampled voxel size of 3 × 3 × 3 mm. Next, spatial smoothing with a 6 mm full-width at half maximum Gaussian kernel, linear detrending, bandpass temporal filtering (0.01–0.1 Hz), and nuisance covariate regression (24 Friston parameters, white matter, cerebrospinal fluid, and global signal) were also performed. The ‘head motion scrubbing’ method proposed by Power and colleagues was used to ensure that motion artifacts did not contribute to the group differences (18). While over 150 volumes were collected for each participant, those volumes with framewise displacement (FD) greater than 0.5 mm were identified and excluded (19). Nodes were defined using the Shen 268-node functional brain atlas, which can be grouped into 10 specific canonical networks that can be used to specify brain networks that contribute in the classification process (20, 21). Functional connectivity was calculated based on the mean time courses of each node (i.e., the average time course of voxels within the node). Next, the functional connectivity matrix was estimated as Pearson’s correlation coefficients of the time series between all pairs of regions. Fisher r-to-z transformation was further applied to convert each correlation coefficient to a z score for normality. Finally, for each subject, a symmetric 268 × 268 resting state functional connectivity (rsFC) matrix was generated in which each element of the matrix represents the functional connectivity strength between two individual nodes (hereafter referred to as an ‘edge’). For both datasets, subjects with excessive head motion (i.e., defined *a priori* as maximal translational or rotational motion parameters >3 mm or 3° during the fMRI scan) were excluded (22–24). Additionally, the data from two participants in Dataset 2 could not successfully be preprocessed and were also excluded from further analysis. Excluded subjects’ identification numbers are in the Supplementary material.

We checked whether the demographic variables between patients and controls significantly differed between groups because age and gender can affect functional connectivity (25, 26). In addition, Dataset 1 included images from two different scanners, which can also affect functional connectivity estimation. Patients and controls in Dataset 1 had significantly different age and gender, and there was a significant difference in scanners used for imaging. To control for these group differences, we used the ‘matchit’ R package (27) to match propensity scores for Dataset 1. Overall, 47 patients and 47 controls from Dataset 1 and 62 patients and 73 controls from Dataset 2 were analyzed.

### Multivariate classification using rsFC

The preprocessed data and analysis code are available at https://osf.io/agqtn/. To predict schizophrenia using rsFC, we used connectome-based predictive modeling (CPM), a data-driven protocol for developing predictive models of brain–behavior relationships (12). We modified the CPM approach by replacing its core learning algorithm with a linear support vector machine (SVM) (28, 29). We briefly explain how the CPM-SVM procedure works. The prediction procedure was as follows: across all subjects in the training set, each edge in the rsFC matrices was Pearson-correlated to the subjects’ group label (i.e., whether each subject is schizophrenia (1) or healthy control (0)). Subsequently, those edges which correlated significantly (*p* < 0.05) with the group label were selected as features. The threshold of 0.05 was determined by optimal threshold exploration. We tested four different *p*-thresholds (0.05, 0.01, 0.005, 0.001) and identified the one that leads to the highest predictive accuracy (30). Considering the sign of the resultant *r* values, edges significantly correlated with group label were divided into increased or decreased rsFC. Then, for each subject, the identified edges were summed into two predictive variables to reduce high dimensionality. Three SVM models were trained and tested, to discriminate which feature set is the most predictive in classifying schizophrenia from healthy controls. We used MATLAB function ‘fitcsvm’ with default options and the box constraint hyperparameter C was 1.

Predicted group label = rsFC matrix increased in schizophrenia.
Predicted group label = rsFC matrix decreased in schizophrenia.
Predicted group label = rsFC matrix increased + decreased in schizophrenia.

**Figure 1:**
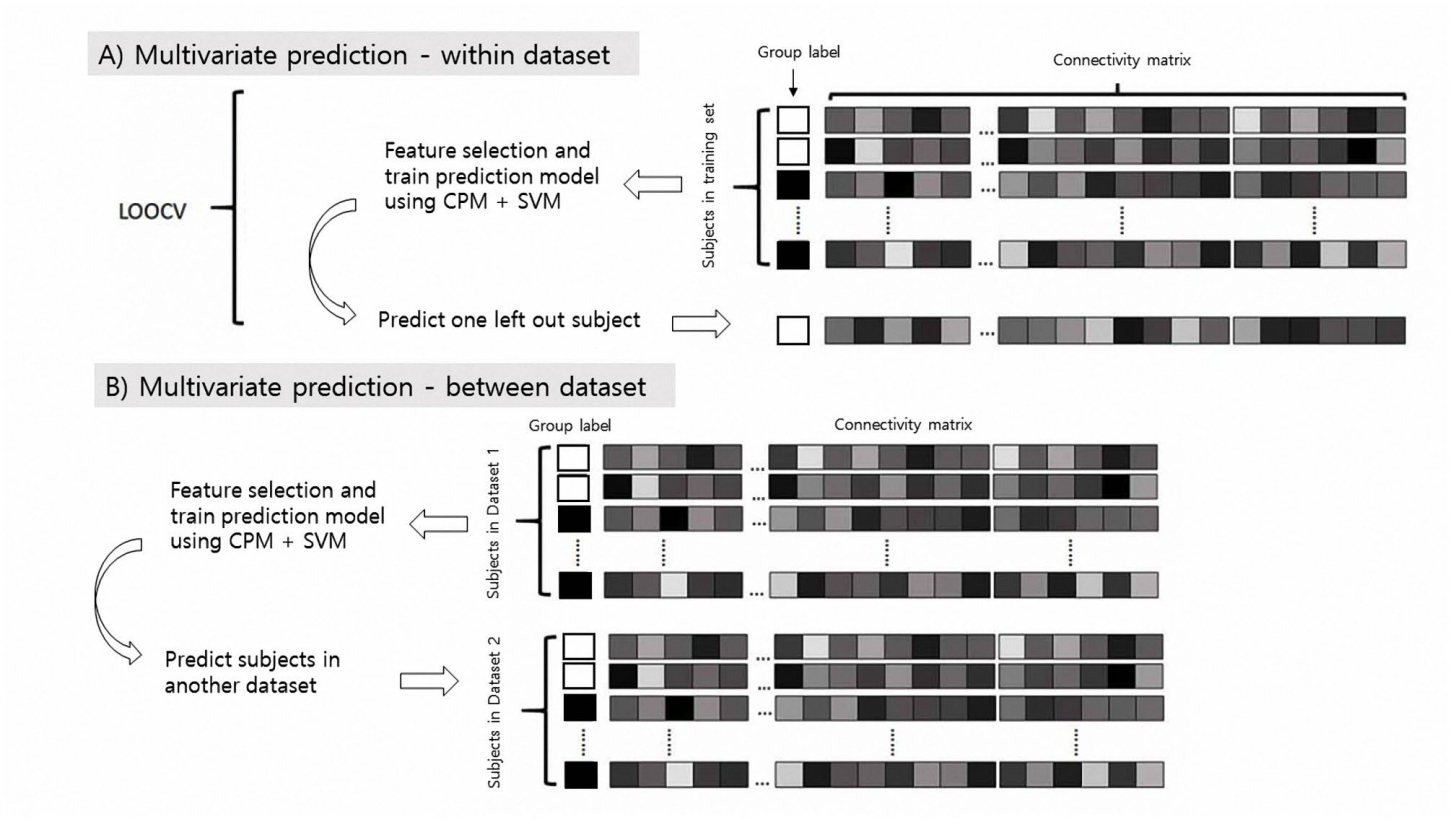
Schematic of multivariate classification using resting-state functional connectivity (rsFC). CPM, connectome-based predictive modeling, SVM, support vector machine.

We conducted within- and between-dataset predictions (Figure 1). For within-dataset prediction, we used a leave-one-subject-out cross-validation (LOOCV) process to protect against overfitting (12), and for between-dataset prediction, one dataset was used as the training set and another was used as the test set, and vice versa. We additionally conducted10-fold cross-validation to validate the model performance. Model performance was assessed mainly using classification accuracy, and we performed both the permutation test and sign test to determine the significance of our model (31). To generate null distributions for permutation testing, we randomly shuffled the group label and reran the modified CPM analysis with the shuffled labels 1,000 times. Based on these null distributions, the *p* values for leave-one-out predictions were calculated (32).

For interpretation purposes, we identified edges selected for the between-dataset prediction model and those edges that appear in 90% of the leave-one-out process to yield ‘consensus edges’ (12). Visualization of the edges was achieved using BioImage Suite.^2^

In addition, although we followed similar preprocessing steps used in previous articles, there is not a single “right” way to process resting state data that reveals the “true” nature of the brain (33). For example, global signal regression may introduce artificial anticorrelations (33). We conducted preprocessing without global signal regression and head motion scrubbing and checked the results are similar.

## Results

### Demographics

The characteristics of the study participants are displayed in Table 1. After propensity matching, the age and gender between the groups in Datasets 1 and 2 showed no significant differences.

**Table 1:** Demographic characteristics of participants.

	Dataset 1 (UCLA)			Dataset 2 (COBRE)		
Variables	SCZ	CON	statistic	SCZ	CON	statistic
Sample size	47	47		62	73	
Age (years)	36.5	36.7	*t* = 0.104, *p* = 0.917	38.4	35.9	*t* = −1.08, *p* = 0.270
Gender (M/F)	34/13	35/12	*χ^2^* = 0.06 *p* = 0.815	48/14	50/23	*χ^2^* = 1.34 *p* = 0.246

### Multivariate classification using rsFC

**Figure 2:**
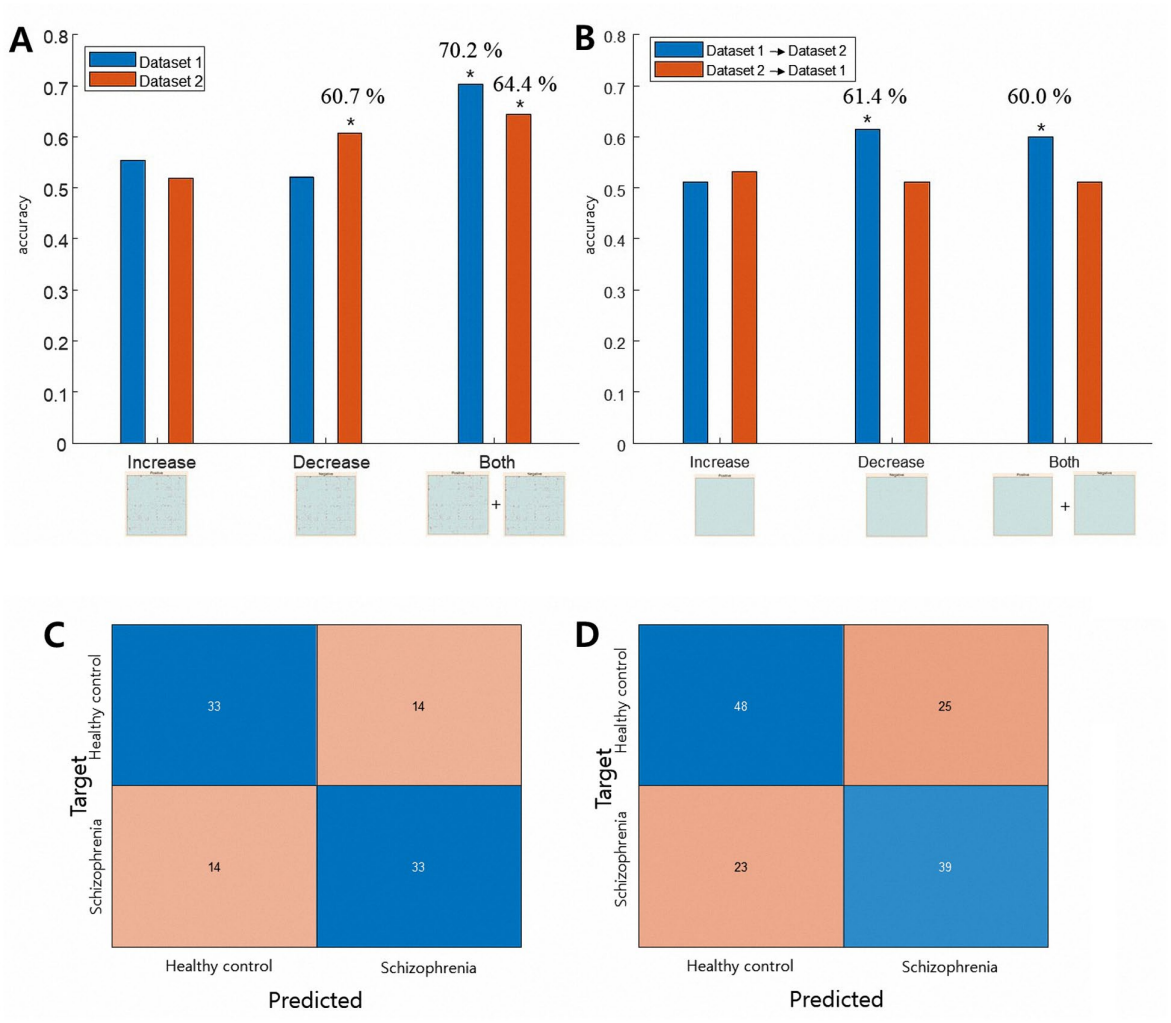
Results of the classification analysis. Panel **A** shows the results of the within-dataset leave-one-out classification. For both datasets, CPM-SVM classification using both increased and decreased edges showed the highest classification accuracy with a permutation test *p* < 0.01 (last two columns). Panel **B** shows the results of the external dataset classification. When Dataset 1 was used as the training set, CPM-SVM classification using the decreased matrix and both matrices could predict subjects in Dataset 2 above chance level. (* in the figure denotes both the permutation test and sign test *p* < 0.02). Panel **C** (UCLA dataset) and **D** (COBRE dataset) shows the confusion matrix of the within-dataset classification result. Blue boxes indicate individuals correctly identified by the model.

Figure 2 summarizes the model performance for individual classification based on increased and decreased rsFC. Notably, for both datasets, the best prediction model was the one that used both increased and decreased rsFC as prediction features (Figure 2, panel A, noted as “Both”). For both datasets, the model accurately identified individuals with schizophrenia above chance (Dataset 1: accuracy = 70.2%, permutation test *p* = 0.001, sign test *p* < 0.001; Dataset 2: accuracy = 64.4%, permutation test *p* = 0.003, sign test *p* = 0.001). In addition, using decreased rsFC in Dataset 2 could predict schizophrenia with an above-chance level accuracy when tested within the dataset (accuracy = 60.7%, permutation *p* = 0.005, sign test *p* = 0.016). Result of 10-fold cross-validation were similar and detailed result is in the Supplementary material. Our sensitivity analysis using different preprocessing steps also showed the both increased and decreased rsFC achieved the best prediction accuracy (Supplementary results).

We also investigated the generalizability of the identified edges between datasets. The between-dataset prediction showed somewhat different results. When the CPM-SVM prediction model was trained with Dataset 1 and tested on Dataset 2 (Figure 2, panel B, blue bars), feature sets of only decreased rsFC could best predict schizophrenia with above chance (decreased rsFC: accuracy = 61.5%, permutation *p* = 0.003, sign test *p* = 0.009; both rsFC: accuracy = 60.0%, permutation *p* = 0.001, sign test *p* = 0.025). None of the CPM-SVM prediction models trained with Dataset 2 and tested on Dataset 1 could make predictions above chance (Figure 2, panel B, red bars).

**Figure 3:**
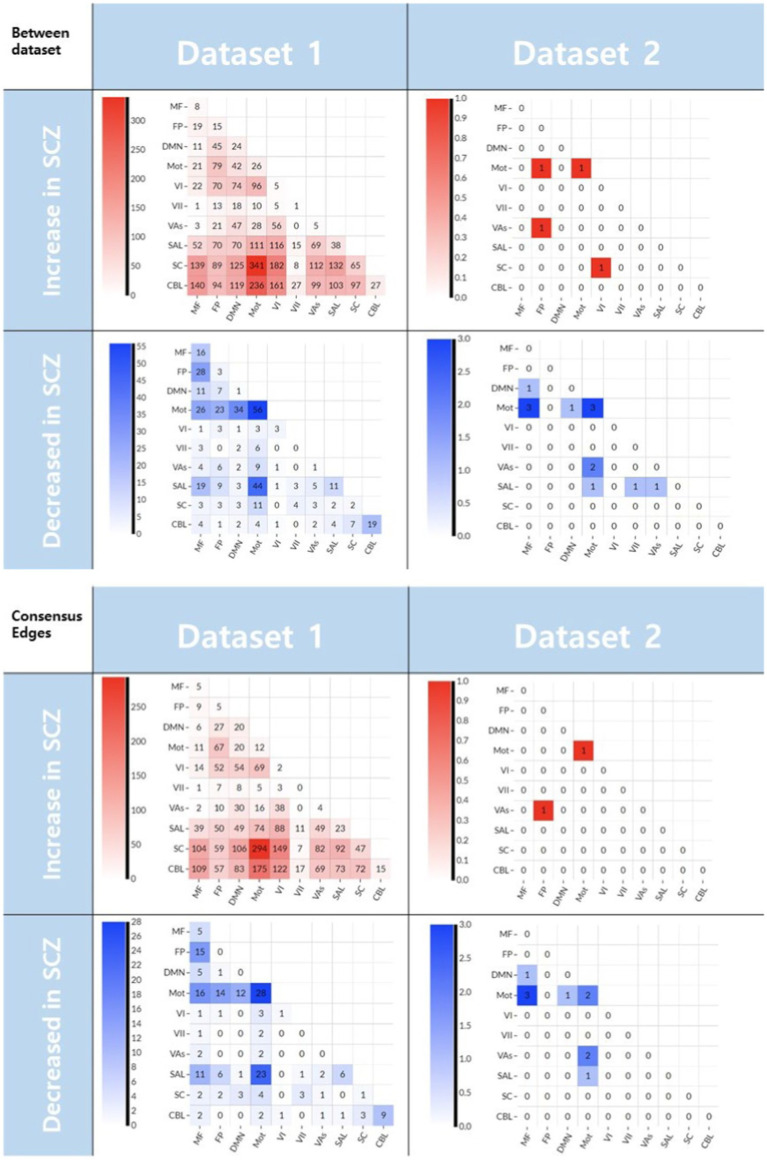
The edges selected in CPM-SVM prediction between datasets (upper) and the ‘consensus edges’ selected during the of the leave-one-out process (lower). The numbers denote the number of edges selected. Those edges that decreased in the schizophrenia patients linked the motor to other networks (blue matrix plots). Meanwhile, the edges that increased in the patient group did not have a similar pattern between the two datasets (red matrix plots). CBL, cerebellum; SC, subcortical; SAL, salience; Vas, visual association; VI, visual A; VII, visual B; Mot, motor; DMN, default mode network; FP, frontoparietal; MF, medial frontal.

For interpretation purposes, the edges selected for the between dataset prediction model and the ‘consensus edges’ in the leave-one-out process are grouped into ten specific functional networks (34). When overlapped with canonical networks, the majority of decreased rsFC were connected within and between the motor networks for both datasets (Figure 3).

## Discussion

In this study, we investigate the effects of different rsFC feature sets (only those increased or decreased in schizophrenia or their combination) on the performance of the CPM – to determine whether the increased connectivity has added information regarding schizophrenia. We found a few key points. First, we noticed that combining both the increased and decreased rsFC achieved the best prediction accuracy for both datasets (Dataset 1: accuracy = 70.2%, permutation *p* = 0.001; Dataset 2: accuracy = 64.4%, permutation *p* = 0.003). Second, when tested across datasets, decreased rsFC in Dataset 1. was well generalized to predict schizophrenia in patients from Dataset 2.

There are two potential applications for multivariate brain decoding: (1) brain decoding for real-world applications, and (2) multivariate hypothesis testing (11, 35). In the first case, a brain decoder with maximum prediction power is desired. In the second case, multivariate decoding is suitable to investigate the information contained in distributed patterns of neural activity to infer the functional role of brain networks (36). In this study, we are interested in the second application of brain decoding which can be considered a multivariate hypothesis testing. Nonetheless, the prediction accuracy we obtained is comparable to previous machine learning classification studies distinguishing patients with schizophrenia from healthy controls. To date, machine learning algorithms with fMRI and structural MRI features have been used in schizophrenia diagnosis, and the performance of machine learning algorithms varied from 70 to 90% in terms of accuracy (37). For example, in a recent study, Lei et al. classified healthy controls and patients with schizophrenia and reported a balanced accuracy of 65.7–73.8% using the same open dataset and deep learning ((17), Table 2, datasets 5 and 6 in the reference article). We achieved 64–76% classification accuracy which is expectable and comparable to previous machine learning studies, considering we did not include higher-order polynomial terms, because our prior goal was to achieve straight forward interpretation of feature weights (12).

When it comes to multivariate hypothesis testing for interpretation, we demonstrated the added value of incorporating both increased and decreased rsFC features into the CPM. If adding a predictor results in an accuracy increase in multivariate analysis, it can be said that the predictor has added information to the outcome variable (13). In addition, incorporating changes to rsFC in both directions to CPM does not guarantee an increase in prediction accuracy (i.e., (30, 38)), supporting that increased rsFC in schizophrenia has complementary information to decreased rsFC regarding schizophrenia. Secondly, it should be noted that only the decreased rsFC in Dataset 1 generalized well to predict schizophrenia in patients from Dataset 2 in the present work. We suggest a few interpretations of this finding. One is these results imply the decreased rsFC is rather universal in schizophrenia while increased rsFC is local. When we grouped the edges detected in the between-dataset prediction into ten canonical functional systems (Figure 3), we recognized the shared distribution of decreased rsFC in the motor network. In a previous article, decreased rsFC in the motor network in schizophrenia was related to the symptomatology of psychosis, and suggested that these networks may be contributing to the etiology of the disease (39). In addition, reduced cortical volume and attenuated activation of the precentral gyrus have been associated with motor-related cognitive dysfunction in schizophrenia (40, 41). However, the distribution of increased rsFC in schizophrenia does not overlap between datasets (Figure 3, upper row). We suggest that this heterogeneity of the increased rsFC in schizophrenia may be the underlying cause of the inconsistent previous reports about the directionality of connectivity alteration in schizophrenia (2–5). These findings can be explained by a previous theory proposed to explain functional connectivity changes in schizophrenia (6). Lynall et al. discovered that schizophrenia patients show a decrease in hubness, resulting in lower regional connectivity strength across the brain, explaining the general decrease in connectivity. Meanwhile, higher regional diversity was also found, and we suggest that this diversity results in rather randomly and inconsistently distributed increased connectivity. In addition, we also suggest global signal regression in preprocessing may contributed to artificial rsFC anticorrelations in both datasets and result in shared decreased rsFC. When we preprocessed the data without global signal regression the generalizability of decreased rsFC between datasets was not significant (Supplementary Figure S2).

There are several limitations in this study. First is data decay. Open data allows researchers to explore pre-existing datasets in new ways. However, if many researchers reuse the same dataset, multiple statistical testing may increase false positives (42). This is not avoidable since we used openly available datasets. Second, the heterogeneity of schizophrenia between the two datasets. Two subjects in the COBRE dataset and 11 subjects in the UCLA dataset had schizoaffective disorder. Third, both of the schizophrenia patients groups were not medication-naïve. Antipsychotic medication may lead to changes in brain function (43), which may have contributed to relative low classification accuracy between datasets.

In conclusion, by using a data-driven and linear multivariate approach, we found evidence that incorporating increased and decreased rsFC has additional information regarding schizophrenia, although decreased rsFC is more universal across the datasets. We suggested our findings can explain the inconsistent discovery of increased rsFC in schizophrenia and give an insight into general distribution pattern of rsFC alteration.

## Data availability statement

The preprocessed data and analysis code presented in the study are publicly available. This data can be found here: https://osf.io/agqtn/.

## Ethics statement

Ethical review and approval was not required in accordance with local legislation.

## Author contributions

MinhK: conceptualization, software, investigation, writing, and funding acquisition. JS and SY: conceptualization, investigation, and writing. MincK: conceptualization, software, investigation, writing, funding acquisition, and visualization. All authors contributed to the article and approved the submitted version.

## Funding

This research was supported by the Hallym University Research Fund, 2022 (HURF-2022-54), and the National Research Foundation of Korea (NRF) grant funded by the Korea government (MSIT) (No. NRF-2022R1F1A1076333).

## Conflict of interest

The authors declare that the research was conducted in the absence of any commercial or financial relationships that could be construed as a potential conflict of interest.

## Publisher’s note

All claims expressed in this article are solely those of the authors and do not necessarily represent those of their affiliated organizations, or those of the publisher, the editors and the reviewers. Any product that may be evaluated in this article, or claim that may be made by its manufacturer, is not guaranteed or endorsed by the publisher.

## Footnotes

^1^http://fcon_1000.projects.nitrc.org/indi/retro/cobre.html

^2^https://www.nitrc.org/projects/bioimagesuite/

## References

[1] Friston KJ. The disconnection hypothesis. Schizophr Res. (1998) 30:115–25. doi: 10.1016/S0920-9964(97)00140-0 9549774

[2] Pettersson-Yeo W Allen P Benetti S Mcguire P Mechelli A. Dysconnectivity in schizophrenia: where are we now? Neurosci Biobehav Rev. (2011) 35:1110–24. doi: 10.1016/j.neubiorev.2010.11.004, PMID: 21115039

[3] Li S Hu N Zhang W Tao B Dai J Gong Y. Dysconnectivity of multiple brain networks in schizophrenia: a meta-analysis of resting-state functional connectivity. Front Psych. (2019) 10:482. doi: 10.3389/fpsyt.2019.00482, PMID: 31354545 PMC6639431

[4] Brandl F Avram M Weise B Shang J Simões B Bertram T. Specific substantial dysconnectivity in schizophrenia: a transdiagnostic multimodal meta-analysis of resting-state functional and structural magnetic resonance imaging studies. Biol Psychiatry. (2019) 85:573–83. doi: 10.1016/j.biopsych.2018.12.003, PMID: 30691673

[5] Dong D Wang Y Chang X Luo C Yao D. Dysfunction of large-scale brain networks in schizophrenia: a meta-analysis of resting-state functional connectivity. Schizophr Bull. (2018) 44:168–81. doi: 10.1093/schbul/sbx034, PMID: 28338943 PMC5767956

[6] Lynall M-E Bassett DS Kerwin R Mckenna PJ Kitzbichler M Muller U. Functional connectivity and brain networks in schizophrenia. J Neurosci. (2010) 30:9477–87. doi: 10.1523/JNEUROSCI.0333-10.2010, PMID: 20631176 PMC2914251

[7] Li X Towe SL Bell RP Jiang R Hall SA Calhoun VD. The individualized prediction of neurocognitive function in people living with HIV based on clinical and multimodal connectome data. IEEE J Biomed Health Inform. (2023) 27:1–11. doi: 10.1109/JBHI.2023.3240508 PMC10387132 37022271

[8] Woo C-W Chang LJ Lindquist MA Wager TD. Building better biomarkers: brain models in translational neuroimaging. Nat Neurosci. (2017) 20:365–77. doi: 10.1038/nn.4478, PMID: 28230847 PMC5988350

[9] Zhang Y Kimberg DY Coslett HB Schwartz MF Wang Z. Multivariate lesion-symptom mapping using support vector regression. Hum Brain Mapp. (2014) 35:5861–76. doi: 10.1002/hbm.22590, PMID: 25044213 PMC4213345

[10] Valente G Castellanos AL Hausfeld L De Martino F Formisano E. Cross-validation and permutations in MVPA: validity of permutation strategies and power of cross-validation schemes. NeuroImage. (2021) 238:118145. doi: 10.1016/j.neuroimage.2021.118145, PMID: 33961999

[11] Hebart MN Baker CI. Deconstructing multivariate decoding for the study of brain function. NeuroImage. (2018) 180:4–18. doi: 10.1016/j.neuroimage.2017.08.005, PMID: 28782682 PMC5797513

[12] Shen X Finn ES Scheinost D Rosenberg MD Chun MM Papademetris X. Using connectome-based predictive modeling to predict individual behavior from brain connectivity. Nat Protoc. (2017) 12:506–18. doi: 10.1038/nprot.2016.178, PMID: 28182017 PMC5526681

[13] Shmueli G Bruce PC Deokar KR Patel NR. Machine learning for business analytics: Concepts, techniques, and applications with analytic solver data mining. Hoboken, New Jersey, USA: John Wiley & Sons (2023).

[14] Poldrack RA Congdon E Triplett W Gorgolewski K Karlsgodt K Mumford J. A phenome-wide examination of neural and cognitive function. Sci Data. (2016) 3:1–12. doi: 10.1038/sdata.2016.110 PMC5139672 27922632

[15] Wang J Wang X Xia M Liao X Evans A He Y. GRETNA: a graph theoretical network analysis toolbox for imaging connectomics. Front Hum Neurosci. (2015) 9:386. doi: 10.3389/fnhum.2015.00386 26175682 PMC4485071

[16] Kim M Seo J. Impulsivity is related to overhasty risk learning in attention-deficit/hyperactivity disorder: a computational psychiatric approach. J Psychiatr Res. (2021) 143:84–90. doi: 10.1016/j.jpsychires.2021.07.044, PMID: 34461353

[17] Lei D Qin K Pinaya WH Young J Van Amelsvoort T Marcelis M. Graph convolutional networks reveal network-level functional dysconnectivity in schizophrenia. Schizophr Bull. (2022) 48:881–92. doi: 10.1093/schbul/sbac047, PMID: 35569019 PMC9212102

[18] Power JD Mitra A Laumann TO Snyder AZ Schlaggar BL Petersen SE. Methods to detect, characterize, and remove motion artifact in resting state fMRI. NeuroImage. (2014) 84:320–41. doi: 10.1016/j.neuroimage.2013.08.048, PMID: 23994314 PMC3849338

[19] Lei D Pinaya WH Young J Van Amelsvoort T Marcelis M Donohoe G. Integrating machining learning and multimodal neuroimaging to detect schizophrenia at the level of the individual. Hum Brain Mapp. (2020) 41:1119–35. doi: 10.1002/hbm.24863 31737978 PMC7268084

[20] Greene AS Gao S Noble S Scheinost D Constable RT. How tasks change whole-brain functional organization to reveal brain-phenotype relationships. Cell Rep. (2020) 32:108066. doi: 10.1016/j.celrep.2020.108066, PMID: 32846124 PMC7469925

[21] Shen X Tokoglu F Papademetris X Constable RT. Groupwise whole-brain parcellation from resting-state fMRI data for network node identification. NeuroImage. (2013) 82:403–15. doi: 10.1016/j.neuroimage.2013.05.081, PMID: 23747961 PMC3759540

[22] Li H Xiong L Xie T Wang Z Li T Zhang H. Incongruent gray matter atrophy and functional connectivity of striatal subregions in behavioral variant frontotemporal dementia. Cereb Cortex. (2022) 33:6103–10. doi: 10.1093/cercor/bhac487 36563002

[23] Ye Y Wang C Lan X Li W Fu L Zhang F. Baseline patterns of resting functional connectivity within posterior default-mode intranetwork associated with remission to antidepressants in major depressive disorder. NeuroImage Clin. (2022) 36:103230. doi: 10.1016/j.nicl.2022.103230, PMID: 36274375 PMC9668631

[24] Zhu J Zhang S Cai H Wang C Yu Y. Common and distinct functional stability abnormalities across three major psychiatric disorders. NeuroImage Clin. (2020) 27:102352. doi: 10.1016/j.nicl.2020.102352, PMID: 32721869 PMC7393318

[25] Geerligs L Renken RJ Saliasi E Maurits NM Lorist MM. A brain-wide study of age-related changes in functional connectivity. Cereb Cortex. (2015) 25:1987–99. doi: 10.1093/cercor/bhu012 24532319

[26] Zhang C Dougherty CC Baum SA White T Michael AM. Functional connectivity predicts gender: evidence for gender differences in resting brain connectivity. Hum Brain Mapp. (2018) 39:1765–76. doi: 10.1002/hbm.23950, PMID: 29322586 PMC6866578

[27] Stuart EA King G Imai K Ho D. MatchIt: nonparametric preprocessing for parametric causal inference. Journal of Statistical Software. (2011) 42.

[28] Kim M Sim S Yang JS Kim M. Multivariate prediction of long COVID headache in adolescents using gray matter structural MRI features. Front Hum Neurosci. (2023) 17:254. doi: 10.3389/fnhum.2023.1202103 PMC10267340 37323930

[29] Song KR Potenza MN Fang XY Gong GL Yao YW Wang ZL. Resting-state connectome-based support-vector-machine predictive modeling of internet gaming disorder. Addict Biol. (2021) 26:e12969. doi: 10.1111/adb.12969 33047425

[30] Ren Z Daker RJ Shi L Sun J Beaty RE Wu X. Connectome-based predictive modeling of creativity anxiety. NeuroImage. (2021) 225:117469. doi: 10.1016/j.neuroimage.2020.117469, PMID: 33099006

[31] Sacchet MD Prasad G Foland-Ross LC Thompson PM Gotlib IH. Support vector machine classification of major depressive disorder using diffusion-weighted neuroimaging and graph theory. Front Psych. (2015) 6:21. doi: 10.3389/fpsyt.2015.00021 PMC4332161 25762941

[32] Pereira F Mitchell T Botvinick M. Machine learning classifiers and fMRI: a tutorial overview. NeuroImage. (2009) 45:S199–209. doi: 10.1016/j.neuroimage.2008.11.007, PMID: 19070668 PMC2892746

[33] Murphy K Fox MD. Towards a consensus regarding global signal regression for resting state functional connectivity MRI. NeuroImage. (2017) 154:169–73. doi: 10.1016/j.neuroimage.2016.11.052, PMID: 27888059 PMC5489207

[34] Finn ES Shen X Scheinost D Rosenberg MD Huang J Chun MM. Functional connectome fingerprinting: identifying individuals using patterns of brain connectivity. Nat Neurosci. (2015) 18:1664–71. doi: 10.1038/nn.4135, PMID: 26457551 PMC5008686

[35] Kia SM Vega Pons S Weisz N Passerini A. Interpretability of multivariate brain maps in linear brain decoding: definition, and heuristic quantification in multivariate analysis of MEG time-locked effects. Front Neurosci. (2017) 10:619. doi: 10.3389/fnins.2016.00619 28167896 PMC5253369

[36] Mahmoudi A Takerkart S Regragui F Boussaoud D Brovelli A. Multivoxel pattern analysis for FMRI data: a review. Comput Math Methods Med. (2012) 2012:1–14. doi: 10.1155/2012/961257, PMID: 23401720 PMC3529504

[37] Guo Y Qiu J Lu W. Support vector machine-based schizophrenia classification using morphological information from amygdaloid and hippocampal subregions. Brain Sci. (2020) 10:562. doi: 10.3390/brainsci10080562, PMID: 32824267 PMC7465509

[38] Antons S Yip SW Lacadie CM Dadashkarimi J Scheinost D Brand M. Connectome-based prediction of craving in gambling disorder and cocaine use disorder. Dialogues Clin Neurosci. (2023) 25:33–42. doi: 10.1080/19585969.2023.2208586, PMID: 37190759 PMC10190201

[39] Bernard JA Goen JR Maldonado T. A case for motor network contributions to schizophrenia symptoms: evidence from resting-state connectivity. Hum Brain Mapp. (2017) 38:4535–45. doi: 10.1002/hbm.23680, PMID: 28603856 PMC5547006

[40] Watanuki T Matsuo K Egashira K Nakashima M Harada K Nakano M. Precentral and inferior prefrontal hypoactivation during facial emotion recognition in patients with schizophrenia: a functional near-infrared spectroscopy study. Schizophr Res. (2016) 170:109–14. doi: 10.1016/j.schres.2015.11.012, PMID: 26612087

[41] Zhou S-Y Suzuki M Hagino H Takahashi T Kawasaki Y Matsui M. Volumetric analysis of sulci/gyri-defined in vivo frontal lobe regions in schizophrenia: precentral gyrus, cingulate gyrus, and prefrontal region. Psychiatry Res Neuroimaging. (2005) 139:127–39. doi: 10.1016/j.pscychresns.2005.05.005 15967647

[42] Thompson WH Wright J Bissett PG Poldrack RA. Dataset decay and the problem of sequential analyses on open datasets. elife. (2020) 9:e53498. doi: 10.7554/eLife.53498, PMID: 32425159 PMC7237204

[43] Vogel T Smieskova R Schmidt A Walter A Harrisberger F Eckert A. Increased superior frontal gyrus activation during working memory processing in psychosis: significant relation to cumulative antipsychotic medication and to negative symptoms. Schizophr Res. (2016) 175:20–6. doi: 10.1016/j.schres.2016.03.033, PMID: 27102424

