# Supplementary material for: Bidirectional connectivity alterations in schizophrenia: a multivariate, machine-learning approach
Source: Front Psychiatry. 2023 Sep 7;14:1232015. doi: 10.3389/fpsyt.2023.1232015 (PMC10512460; doi:10.3389/fpsyt.2023.1232015)
Supplement: Supplementary file 1 [file Data_Sheet_1.docx]

**Supplementary material**

Excluded subjects’ IDs and corresponding reason

Datatset 1

- excessive head motion : sub-10269, sub-10271, sub-10339, sub-10455, sub-10934, sub-10998, sub-50008, sub-50020, sub-50022

Dataset 2

- excessive head motion : 0040001, 0040006, 0040010, 0040045, 0040060, 0040073, 0040079, 0040099
- disenrolled : 0040070, 00400483
- processing fail : 0040072, 0040089

**Supplementary results**

*Multivariate classification using 10-fold cross-validation*

While leave-one-out cross-validation is suggested for small (n<200) sample sizes, the small sample sizes can still lead to overfitting. Complementarily, 5- or 10-fold cross-validation represents a good compromise between model bias and variance (Scheinost et al., 2019).

Supplementary Figure 1 summarizes the model performance for individual classification by 10-fold cross-validation. We performed 100 iterations of 10-fold cross-validation. Notably, the prediction performance for both datasets is similar to leave-one-out cross-validation. The best prediction model was the one that used both increased and decreased rsFC as prediction features.

*Sensitivity analysis using different preprocessing steps*

Supplementary Figure 2, 3 and supplementary table 1 summarizes the sensitivity analysis regarding preprocessing steps. In general, our original finding was replicated. Regardless of preprocessing steps, the CPM-SVM model utilizing both increased and decreased rsFC achieved the best prediction accuracy for both datasets. When tested between datasets, the result was slightly different: not doing global signal regression made the decreased connectivity not generalize significantly.

**Supplementary Figure 1.** The model performance for individual classification by 10-fold cross-validation. We performed 100 iterations of 10-fold cross-validation. The number in parenthesis denoted the mean classification accuracy of 100 iteration.


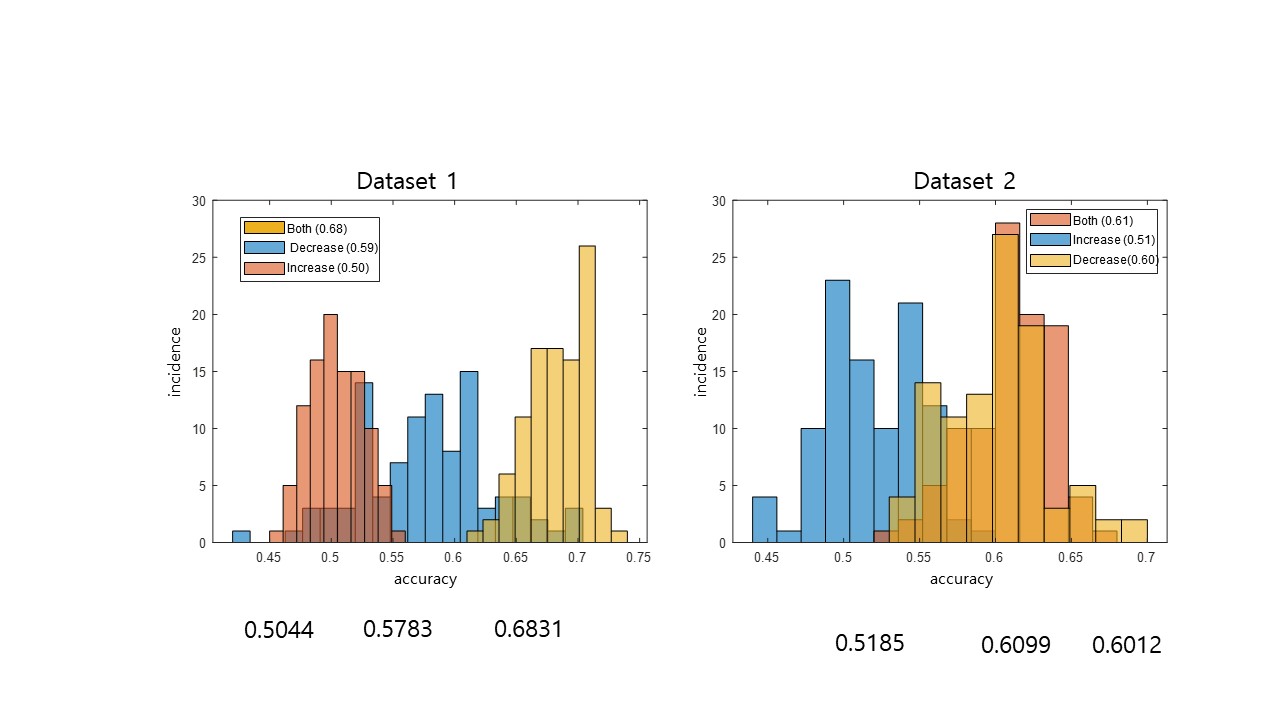


**Supplementary Figure 2.** Results of the classification analysis using preprocessed rsFC without global signal regression. Panel A shows the results of the within-dataset leave-one-out classification. For both datasets, CPM-SVM classification using both increased and decreased edges showed the highest classification accuracy with a sign test *p* <0.01 (last two columns). Panel B shows the results of the external dataset classification. When Dataset 1 was used as the training set, CPM-SVM classification using the both matrices could predict subjects in Dataset 2 above chance level. (* in the figure denotes the sign test *p* <0.05). Panel C (UCLA dataset) and D (COBRE dataset) shows the boxplot of rsFC distribution before and after global signal regression (GSR). Interestingly, the rsFC outliers were decreased after GSR in COBRE dataset.


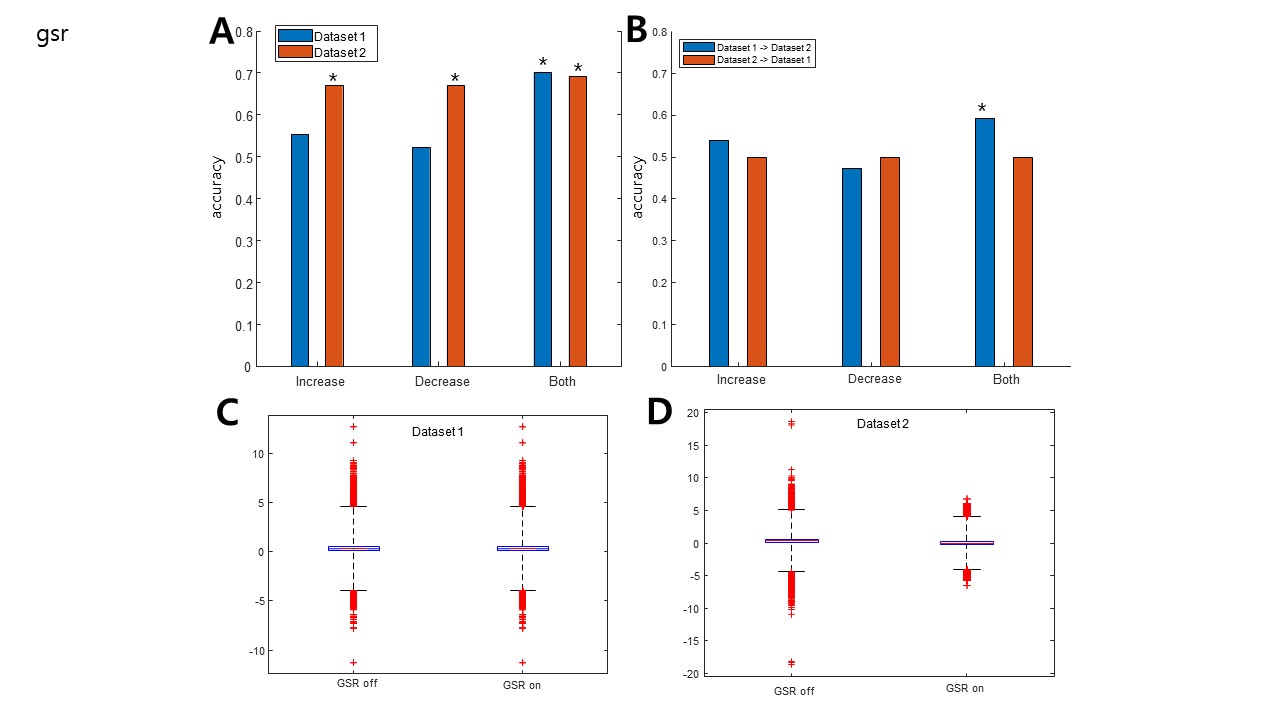


**Supplementary Figure 3.** Results of the classification analysis using preprocessed rsFC without scrubbing. Panel A shows the results of the within-dataset leave-one-out classification. For both datasets, CPM-SVM classification using both increased and decreased edges showed the highest classification accuracy with a sign test *p* <0.01 (last two columns). Panel B shows the results of the external dataset classification. When Dataset 1 was used as the training set, CPM-SVM classification using the all three matrices could predict subjects in Dataset 2 above chance level. (* in the figure denotes the sign test *p* <0.05). Panel C (UCLA dataset) and D (COBRE dataset) shows the boxplot of rsFC distribution before and after head motion scrubbing. Interestingly, the rsFC outliers were increased after head motion scrubbing for the two datasets.


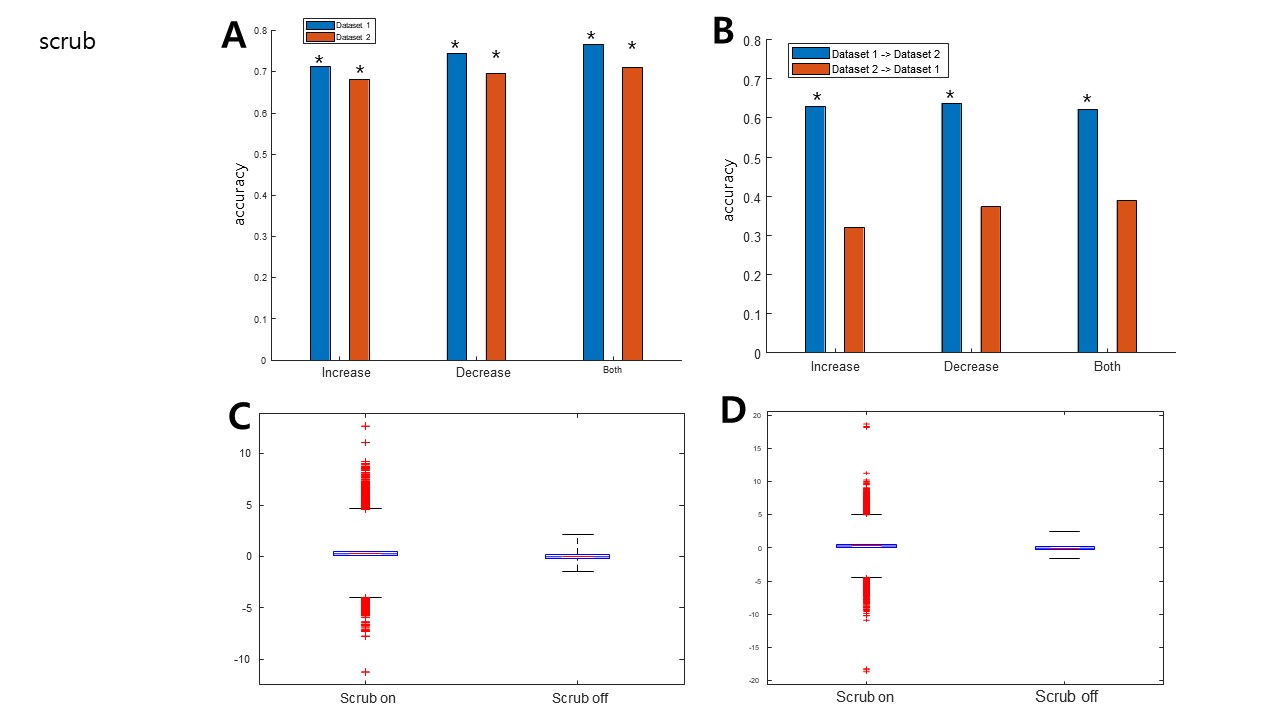


**Supplementary Table 1.** Classification accuracy and corresponding *p*-value of according to preprocessing methods. Significant prediction accuracies are in bold.

| Original | **Within Dataset** | | | | **Dataset1🡪2** | |
| --- | --- | --- | --- | --- | --- | --- |
|  | **Dataset 1** | | **Dataset 2** | |  |  |
|  | Classification accuracy | *p*-value | Classification accuracy | *p*-value | Classification accuracy | *p*-value |
| **Increase** | 0.55 | 0.35 | 0.52 | 0.73 | 0.51 | 0.86 |
| **Decrease** | 0.52 | 0.75 | **0.60** | 0.02 | **0.61** | <0.01 |
| **Both** | **0.70** | <0.01 | **0.64** | <0.01 | **0.60** | 0.02 |

| Without  global signal regression | **Within Dataset** | | | | **Dataset1🡪2** | |
| --- | --- | --- | --- | --- | --- | --- |
|  | **Dataset 1** | | **Dataset 2** | |  |  |
|  | Classification accuracy | *p*-value | Classification accuracy | *p*-value | Classification accuracy | *p*-value |
| **Increase** | 0.55 | 0.35 | **0.66** | <0.01 | 0.52 | 0.60 |
| **Decrease** | 0.52 | 0.75 | **0.66** | <0.01 | 0.47 | 0.60 |
| **Both** | **0.70** | <0.01 | **0.69** | <0.01 | **0.58** | 0.05 |

| Without  scrubbing | **Within Dataset** | | | | **Dataset1🡪2** | |
| --- | --- | --- | --- | --- | --- | --- |
|  | **Dataset 1** | | **Dataset 2** | |  |  |
|  | Classification accuracy | *p*-value | Classification accuracy | *p*-value | Classification accuracy | *p*-value |
| **Increase** | **0.71** | <0.01 | **0.68** | <0.01 | **0.62** | <0.01 |
| **Decrease** | **0.74** | <0.01 | **0.69** | <0.01 | **0.63** | <0.01 |
| **Both** | **0.76** | <0.01 | **0.71** | <0.01 | **0.62** | <0.01 |

**References**

Scheinost, D., Noble, S., Horien, C., Greene, A.S., Lake, E.M., Salehi, M., Gao, S., Shen, X., O'connor, D., and Barron, D.S. (2019). Ten simple rules for predictive modeling of individual differences in neuroimaging. *Neuroimage* 193**,** 35-45.
